# Preservatives from food—For food: Pea protein hydrolysate as a novel bio‐preservative against *Escherichia coli* O157:H7 on a lettuce leaf

**DOI:** 10.1002/fsn3.2489

**Published:** 2021-09-08

**Authors:** Niamh M. Mohan, Amine Zorgani, Leah Earley, Sweeny Chauhan, Sanja Trajkovic, John Savage, Alessandro Adelfio, Nora Khaldi, Marta Martins

**Affiliations:** ^1^ Department of Microbiology Moyne Institute of Preventive Medicine School of Genetics and Microbiology Trinity College Dublin The University of Dublin Dublin Ireland; ^2^ Nuritas Limited Dublin Ireland

**Keywords:** bio‐preservation, *E. coli* O157:H7, food preservation, *Pisum sativum*, protein hydrolysates

## Abstract

Fresh‐cut fruits and vegetables are becoming particularly popular as healthy fast‐food options; however, they present challenges such as accelerated rates of decay and increased risk for contamination when compared to whole produce. Given that food safety must remain paramount for producers and manufacturers, research into novel, natural food preservation solutions which can help to ensure food safety and protect against spoilage is on the rise. In this work, we investigated the potential of using a novel protein hydrolysate, produced by the enzymatic hydrolysis of *Pisum sativum* (PSH), as a novel bio‐preservative and its abilities to reduce populations of *Escherichia coli* O157:H7 after inoculation on a lettuce leaf. While unhydrolyzed *P. sativum* proteins show no antimicrobial activity, once digested, and purified, the enzymatically released peptides induced in vitro bactericidal effects on the foodborne pathogen at 8 mg/ml. When applied on an infected lettuce leaf, the PSH significantly reduced the number of bacteria recovered after 2 hr of treatment. PSH may be preferred over other preservation strategies based on its natural, inexpensive, sustainable source, environmentally friendly process, nontoxic nature, good batch to batch consistency, and ability to significantly reduce counts of *E. coli* both in vitro and in a lettuce leaf.

## Graphical abstract

In this work, we investigated the potential to use *Pisum sativum* hydrolysate (PSH) as a novel bio‐preservative and its abilities to reduce populations of *Escherichia coli* O157:H7 in a lettuce leaf model. PSH may be preferred over other preservation strategies based on its natural, inexpensive, sustainable source, environmentally friendly process, nontoxic nature, good batch to batch consistency and ability to significantly reduce counts of *E. coli* both in vitro and in a lettuce leaf model.

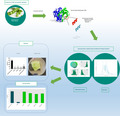

## 1 INTRODUCTION

Increasingly health‐conscious consumers are changing their buying habits. With convenience and nutritional value of paramount importance, products such as bagged lettuce and salads have become increasingly popular in the last 10 years as “healthy fast food” (Koukkidis & Freestone, 2018; Rekhy & McConchie, 2014). The pH of lettuce (5.5–6.0) paired with its high water activity (*a_w_*) value provides optimal conditions for microbial growth (Tirpanalan et al., 2011). This is made worse in cut/shredded lettuce as the surface area is vastly increased making it a highly perishable food which is often implicated in outbreaks of foodborne illness (Qadri et al., 2015). Therefore, in order to avoid these incidents, preservation and processing strategies have been implemented by the food industry to serve two purposes; firstly, to delay enzymatic decay and oxidation which contributes to natural spoilage; and secondly, to control the proliferation of undesirable, pathogenic micro‐organisms that can cause adverse organoleptic changes and often result in food poisoning.

Shiga‐toxin producing *Escherichia coli* O157:H7 poses one of the biggest threats as it is highly virulent with a reported infectious dose of only 10–100 cells (Greig et al., 2010). In 2018, romaine lettuce contaminated with *E. coli* O157:H7 was responsible for a multistate outbreak of food poisoning infecting 210 people, 12% of whom developed hemolytic uremic syndrome (HUS) after hospitalization (CDC, 2018). More recently, in early 2019, *E. coli* O157:H7‐contaminated lettuce grown in California caused 62 illnesses over 16 states (CDC, 2018). As technology in detection, surveillance, and reporting develops, the management of these type of infections has indeed improved (Al‐Qadiri et al., 2006). However, these very recent outbreaks highlight that current microbial control strategies are not always effective at eliminating the bacterial risks encountered from farm to fork.

Bacterial pathogens are commonly spread to lettuce and other vegetables when fields are irrigated with contaminated water. Processing techniques are therefore employed postharvest to reduce pathogenic populations and extend the shelf life of processed products. Physical preservation treatments such as modified atmosphere packaging (Siroli, 2014), edible coatings (Corbo et al., 2015), and irradiation (Misra et al., 2011; Rico et al., 2007) have all been investigated to date and may indeed be employed as hurdle technologies; however, minimal processing such as washes is still favored for lettuce. Washing with household water alone has proven insufficient as a means to reduce pathogenic populations on lettuce leaves (Uhlig et al., 2017). Chlorine is the most common chemical added to wash water to decontaminate fresh‐cut product due to its efficacy, solubility, ease of use, and inexpensive cost (Petri et al., 2015). While concentrations of 200 parts per million (ppm) in water are generally regarded as safe, various authors have expressed concerns regarding the risks associated with carcinogenic by‐products of chlorine, for both public health and the environment, when bound to organic matter such as chloroform, haloketones, and trihalomethane (Abadias et al., 2011; Rico et al., 2007). In addition, fresh‐cut products have high organic loads which subsequently leads to rapid chlorine consumption and greater potential for contamination. These types of products may therefore benefit from combinatory decontamination solutions. Various other washing treatments have been proposed as safe and effective such as hydrogen peroxide, ozone, electrolyzed water, and organic acids. While some have been employed as surface decontaminants on packaging materials, none are currently permitted for use with food (Ölmez, 2010; Rico et al., 2007; Siroli, 2014). Peracetic acid is among one of the most effective methods for controlling microbial contamination in fresh produce and meat products; however, those working closely with the chemical can be adversely affected by prolonged exposure to the acid. An overview of the advantages and disadvantages of current washing preservation methods are illustrated in Table S1.

As consumer demand for natural ingredients and clean labels grows, it is likely that industry will move away from chemical means of food preservation and adopt other alternatives, such as food‐derived antimicrobial peptides and hydrolyzed proteins (Carocho et al., 2015).

Protein plays a critical role in providing the body with essential amino acids for basic nutrition and energy. Additionally, proteins can be a source of physiologically active compounds and encrypted bioactive peptides which are contained within their amino acid sequence (Chalamaiah et al., 2018). These peptides remain “dormant” when within the parent protein and however can exhibit vast bioactivities when cleaved by acid treatment, protease addition, microbial fermentations, or during food digestion by the action of gastric enzymes (Bhat et al., 2015; Cesar Lemes et al., 2016; Garcia et al., 2013; Mora et al., 2019). Given the body's natural biological effort to break down macromolecules into smaller absorbable units, protein hydrolysis, and the production and uptake of bioactive peptides, has occurred for hundreds of years before any functionalities were established (Fitzgerald et al., 2005; Sato et al., 2013). A prominent example is the plethora of bioactive peptides created post–enzymatic hydrolysis and digestion of intact breast milk proteins in an infant's stomach. When compared to whole proteins, protein hydrolysates that contain a complex mixture of peptides are more digestible and bioavailable. Further, these hydrolysates have been widely reported to have anticancer, antihypertensive, antioxidant, opiate, antimicrobial, and immunomodulatory activities (Bhat et al., 2015; Chalamaiah et al., 2018; Nasri, 2017). Enzymatically hydrolyzing proteins outside of the gastrointestinal (GI) tract allows for tighter control of the peptides released. These can then be studied individually or as a mixture for biological activities and potential uses which differ to those of the parent protein. Computer‐assisted approaches for predicting the location of bioactive peptides within known parent protein sequence have been instrumental in changing the way in which peptides are discovered from biological sources.

Aside from those bacterially derived, animal proteins, predominantly those from milk (León‐calvijo et al., 2015), eggs (Mine et al., 2004), meat (Di Bernardini et al., 2011), and skin (Nalinanon et al., 2011), have been exploited as sources of commercial protein hydrolysates. Numerous studies report the activity that bioactive peptides exert when they are released from their precursor proteins. These functions are different from the ones they exert when they are in their intact form (Mine et al., 2004; Slizyte et al., 2016). Except for a selected few, such as soy and rice, plants and pulses have been largely unexplored as sources of protein hydrolysates. This is reiterated by the number of peptide entries in the Database of Antimicrobial Activity and Structure of Peptides (DBAASP) in 2021, where 2,281 peptides are recorded from the animal kingdom, compared with just 273 from the plant kingdom (https://dbaasp.org). In addition to the micronutrients, phytochemicals, and vitamins which give rise to the health promoting effects associated with a plant food rich diet, bioactive peptides have an important part to play in a host of biological functions (López‐Barrios et al., 2014). Focusing specifically on antimicrobial activities, the extent, and strain specificity of the effects largely depends on the amino acid composition, structure, length, and conformation of the individual peptides contained within the hydrolysate (Pane et al., 2017). Cationic peptides are the most prominent type and exert antimicrobial effects in a two‐step fashion. Firstly, the positively charged peptides bind via electrostatic interactions to the negatively charged components of the bacterial cell membrane. Afterward, they form pores and ultimately destroy the integrity of the cell (Mohan et al., 2019). Cationic peptides have been associated with a reduced propensity for causing antimicrobial resistance and therefore offer an important alternative to antibiotics, in the current scenario. While individual synthetically produced peptides may face regulatory and economic hurdles as food preservatives, protein hydrolysates help to overcome these limitations and deliver bioactive peptides in a natural, cost‐effective way which is more favorable in the industrial setting.

In this study, we aim to add knowledge around the untapped potential of plants as sources of bioactive hydrolysates and expand on the predominantly animal based research. In addition, we highlight how an in silico data mining approach paired with protein hydrolysis and mass spectrometry can be used to successfully identify potent protein fragments from plant sources. Specifically, we explore *Pisum sativum* protein hydrolysate (PSH) as a bio‐preservative to inhibit the growth of *E. coli* O157:H7 in an infected lettuce leaf. The fit with plant‐based trends, paired with their inexpensiveness, voluminous production capabilities, natural origins, and history of safe consumption, makes plant protein hydrolysates interesting candidates as alternatives to chemical preservatives with a “from food—for food” approach (Chakrabarti et al., 2014; Hou et al., 2017; Schaafsma, 2009; Zambrowicz et al., 2012).

## 2 MATERIALS AND METHODS

### 2.1 Plant material and reagents

Eighty percent protein powder of *Pisum sativum* was purchased from a commercial supplier. Leucidal liquid was purchased from Active Micro Technologies and used as a comparative control in this study. Leucidal Liquid is a fermented radish hydrolysate bio‐preservative based on an antimicrobial peptide originally derived from the lactic acid bacteria, *Leuconostoc kimchi*. It is an Ecocert‐approved ingredient in certified organic cosmetics and is also on the Whole Foods Acceptable Premium Preservative List (Active Micro Technologies, 2020). Given its antimicrobial application in food, its natural source, and its hydrolyzed protein content, Leucidal Liquid was considered as the most relevant commercially available comparison to PSH. The antimicrobial activity of Leucidal Liquid against various Gram‐negative and Gram‐positive pathogens is available online and so this was used in the infected leaf model as a comparative benchmark (Active Micro Technologies, 2020). Vivaspin 500 Centrifugal Concentrator molecular weight cut off filters (MWCO) were purchased from Sigma Aldrich. Oasis HLB 10 mg sorbent Solid Phase Extraction (SPE) cartridges were obtained from Waters. All bacterial growth media were purchased from Oxoid Sparks Lab Suppliers. Protein content in the samples was determined using Pierce Bicinchoninic Acid Assay (BCA) protein concentration kit, Thermo Fisher Scientific. Formic acid, Optima water, and acetonitrile were all obtained from Fisher Scientific.

### 2.2 Bacterial culture preparation and growth conditions

*Escherichia coli* NCTC 12900 (Serotype O157:H7, verocytotoxin/shiga‐toxin negative) was gifted by the Department of Food Science and Environmental Health, Dublin Institute of Technology, Ireland. *E. coli* cultures were maintained at −80°C in 15% glycerol. *Escherichia coli* was grown in Mueller Hinton broth (MHB) medium. Cultures were prepared by inoculating 10 ml of the selected media with bacteria and incubating overnight for 18 hr at 37°C with agitation. A subculture was prepared from the bacterial suspension by diluting it to an optical density at 600 nm (OD_600_) of ~0.05 in fresh media and reincubating under growth optimal conditions for 2–3 hr until logarithmic phase was reached (OD_600_ ~ 0.5). Bacterial cultures were then adjusted to the desired concentration for assay in phosphate buffer without salt (PBNS). THP‐1 cells were acquired from the American Type Culture Collection (ATCC).

### 2.3 Lettuce leaf preparation

A fresh head of iceberg lettuce (*Lactuca sativa* var. *capitata*) grown in Spain was purchased at a local supermarket in Dublin, Ireland, for each experiment. The lettuce was stored at 4°C and used within three hours of purchase. The outer leaves of the lettuce were removed and discarded. The remaining leaves were cut into 3 x 3 cm^2^ pieces using a sterile knife. The leaves were dipped for 10 s in 70% ethanol before being submerged into sterile water for a further 10 s. Afterward, the lettuce pieces were inoculated with 500 µl of *E. coli* at ~1.1 x 10^4^ cells/ml. The leaves were dried at room temperature in a biosafety cabinet for 15 min to facilitate bacterial adherence.

### 2.4 Hydrolysis of *Pisum sativum* protein powder and preparation of *Pisum sativum* hydrolysate

Protein hydrolysis and drying were carried out according to a method adapted from Aluko, 2018; Kennedy et al., 2020 and Rein et al., 2019. In brief, *P. sativum* protein powder (<80% organic pea powder) was mixed with nonphosphate buffer to raise pH and alkalinity before hydrolysis with food grade serine protease under constant agitation in pH and temperature regulated conditions. After homogenization, the pH was lowered to pH 6 and hydrolysis was performed by the addition of protease for several hours under enzyme‐specific conditions. The hydrolysis procedure was terminated by denaturing the enzymes at 85°C for 15 min in a water bath. Afterward, the samples were cooled; the soluble fraction was separated by centrifugation and further purified by SPE. Finally, the samples were stored at 4˚C for mass spectrometry analysis and bioactivity testing.

### 2.5 Solid‐phase extraction of *Pisum sativum* hydrolysate

The PSH was prepared to a concentration of 10 mg/ml and acidified by directly adding formic acid (reagent grade, Fisher Chemical) to a final concentration of 0.1%. The extraction cartridges were then placed in the vacuum manifold and the vacuum switched on with the main tap off. Two milliliters of acetonitrile (Optima grade, Fisher Chemical) was added to each cartridge, 1 ml at a time allowing the cartridge to empty 80% of the liquid before the next addition. Two milliliters of formic acid dissolved in water was added in the same way. The acidified sample was loaded at gravity flow of 1 ml/min. The cartridges were washed with 4x the sample volume loaded using 0.1% formic acid, then with optima water, 1 ml at a time, respectively. After the final wash, the volume was increased to 15 mm‐Hg and the cartridge was dried out. The vacuum tap was closed and released using the emergency valve. The waste tubes were replaced with 1.5‐ml tubes for sample collection. With the vacuum valve closed, the samples were eluted with 60% acetonitrile at half the sample volume loaded at gravity flow. Once dry, the vacuum was released with the emergency valve prior to removing the samples. After elution, the sample tubes were placed into a SpeedVac and dried overnight. Each 1.5‐ml sample tube was resuspended in 70 µl of Optima water before testing.

### 2.6 Bicinchoninic acid protein determination assay

A bovine serum albumin (BSA) stock solution of 2 mg/ml was prepared by adding 10 mg of BSA into 5 ml of deionized water. Following this, protein standards of 0–2,000 µg/ml were prepared using deionized water according to the manufacturer's instructions. Protein hydrolysates samples were diluted in deionized water to a dilution factor of 10 and 20 to ensure the values would fall within the linear range of the standard curve. Copper II solution and bicinchoninic acid were mixed in a ratio of 1:50, respectively. Following this, 25 µl of the standards and diluted samples were added in triplicate onto a 96‐well plate. Once completed, 200 µl of the preprepared BCA working reagent was added to the standards and samples and the plate was incubated at 37°C for 30 min. After incubation, the OD was read at 562 nm in a microplate spectrophotometer (Synergy H1, BioTek). The protein/peptide concentration was determined by interpolation of the readings of the samples into the equation of the standard curve using the Synergy H1 software.

### 2.7 Mass Spectrometry (MS) analysis of *Pisum sativum* hydrolysate samples

Firstly, PSH samples were filtered through 10 kDa MWCO filters to remove the enzymes used during hydrolysis and any insoluble fractions (Pasupuleki & Braun, 2010). The filtered samples were then further cleaned by running them through SPE cartridges (previously described above; Section 2.5) before being spray dried. Samples were reconstituted in Optima water to assess peptide concentration *via* the BCA assay (detailed in Section 2.6). Liquid chromatography (LC) was performed using a 60 min gradient from 5% to 75% acetonitrile on an EasySpray reverse‐phase column (ES804; Thermo Fisher Scientific) coupled to a Q‐Exactive mass spectrometer (Thermo Fisher). Peptides were loaded on a trapping column and eluted over a 15‐cm analytical column with a 1‐hr gradient at a flow rate of 300 nl/min. The mass spectrometer was operated in data‐dependent mode, with MS and MS/MS performed in the Orbitrap at 70,000 Full width at half maximum (FWHM) and 17,500 FWHM resolution, respectively. From the MS scan, the fifteen most intense ions were selected for MS/MS. Fragmentation spectra from putative peptides collected by data‐dependent acquisition were used for peptide identification. Relative quantification was performed by integrating precursor signal intensities using the PEAKS software (Ma et al., 2003).

### 2.8 Antibacterial activity of *Pisum sativum* hydrolysate against *E. coli* O157:H7

The complete elimination (CE) method was primarily used as the antimicrobial susceptibility testing method. This was performed to avoid any interference of cations within the test media on the bioactivity of the protein hydrolysate. The assay was performed according to Chen et al., (2003) with minor modifications (Chen et al., 2003). Briefly, *E. coli* O157:H7 was grown in liquid broth to mid exponential phase. PBNS was used to make serial dilutions of bacteria at a density of 1.0 x 10^4^ and 1.0 x 10^5^ cells/ml in a 96‐well plate. *Pisum sativum* hydrolysate was added to the wells at the desired concentrations and made up to a final volume of 100 µl. PBNS alone was added to the bacterial suspension as an untreated control. The 96‐well plate was incubated for 24 hr at 37°C. After challenge, 10 µl from each well was spotted onto Mueller Hinton agar in triplicate. This was replicated on three independent days. The spots were left to dry, and the plates were incubated inverted. The first concentration to inhibit bacterial growth was determined as the CE concentration. Surviving colonies at sub‐CE concentrations were counted after 18‐hr incubation at 37°C. The reduction in colony‐forming units (CFU) was assessed relative to the untreated control, and the log reduction induced by the hydrolysate treatment was calculated.

### 2.9 The effects of PSH at inhibiting the growth of *E. coli* O157:H7 in an infected lettuce leaf—stamp on agar method

The lettuce was prepared, inoculated, and dried according to Section 2.3. After this time, 100 µl of PSH was spotted onto the underside of a single leaf piece and spread onto the surface with a sterile cotton swab. PSH was applied to three separate leaves at the concentration previously determined to induce bactericidal effects in vitro. Leucidal liquid was used as a comparative control. PBNS was used as a negative control, as it was the buffer solution for the PSH samples. The treated leaves were placed in sterile petri dishes and refrigerated at 4°C for 2 hr. After this time, the leaves were pressed gently onto agar using sterile forceps. The plates were then inverted and incubated at 37°C for 24 hr. The colonies which grew from the stamped area were counted the following day. PSH‐treated samples were compared with the untreated control.

### 2.10 Toxicity assay

The toxicity of the PSH was evaluated using the thiazolyl blue tetrazolium bromide method (MTT, Sigma) in a cellular viability with human THP‐1 differentiated macrophages (Silva et al., 2016). Macrophages were grown in Roswell Park Memorial Institute (RPMI) medium supplemented with 10% of heat‐inactivated fetal bovine serum (FBS), 1% L‐glutamine, and 1% penicillin‐streptomycin. When the cells reached a confluency of 90%, the cells were pelleted via centrifugation and diluted to a 1:5 ratio with complete RPMI. Differentiation of the monocytes was performed by adding 20 ng/ml phorbol 12‐myristate 13‐acetate (PMA) to cells before seeding in a 96‐well plate at a density of 1.0 x 10^4^ cells/well. The plate was incubated with 5% CO_2_ for 72 hr at 37°C. Following this, cells were treated with increasing concentrations (0.01, 0.1, 1, 10 mg/ml) of PSH before reincubation for 24 hr. After PSH treatment, the media was removed and replaced with 110 µl of MTT solution. This solution was added to each well at a final concentration of 500 µg/ml and cells incubated at 5% CO_2_ for 2 hr at 37°C. After incubation, the media was removed and replaced with of 110 µl of 100% Dimethyl sulfoxide (DMSO) in each well at room temperature. The plate was covered in tin foil and gently agitated on a plate shaker for 5 min to encourage the dissolution of formazan crystals. The OD was measured at 570 nm in a microplate spectrophotometer (Synergy H1, BioTek). Maximum toxicity was determined by cells incubated with 100% DMSO. Cell viability was calculated as a percentage of the untreated control cells. Three technical replicates were performed each day on three independent days.

### 2.11 Statistical analysis

All data are presented as mean ± standard deviation. Replicate numbers for each experiment are indicated in figure legends. Results of in vitro experiments were analyzed by one‐way ANOVA. Statistical significance was defined as * *p* < .05, ** *p* ≤ .01 and *** *p* ≤ .001.

## 3 RESULTS

### 3.1 High performance liquid chromatography—size exclusion chromatograms of hydrolyzed and unhydrolyzed PSH

**FIGURE 1:**
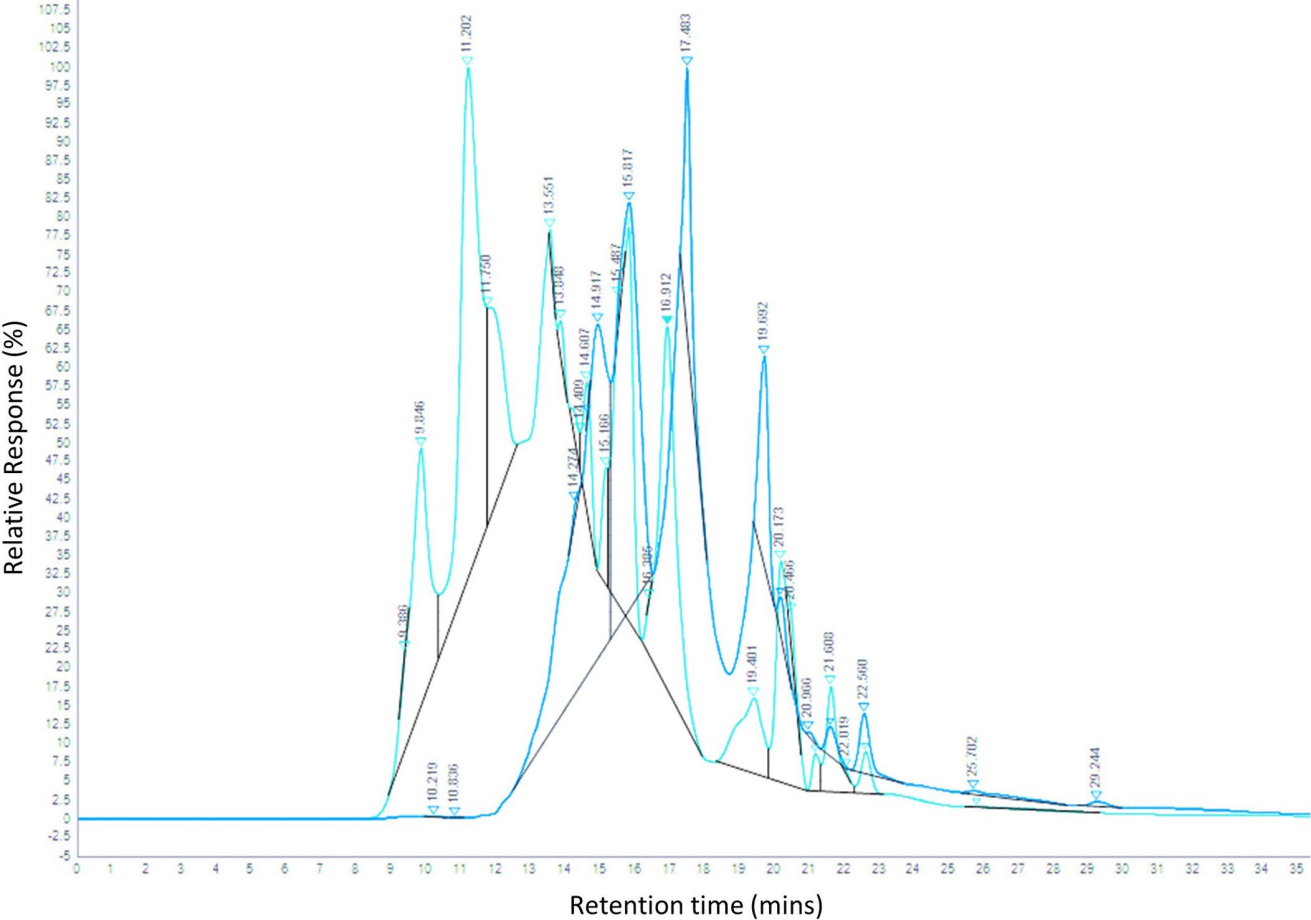
Size exclusion chromatography—high performance liquid chromatography (HPLC) of unhydrolyzed *Pisum sativum* overlaid on PSH. Molecular weight chromatograms of both *P. sativum* materials pre‐ and post–enzymatic digestion. The unhydrolyzed sample has a shorter retention time (9–18 min) while the hydrolyzed PSH eludes later (14–21 min). Unhydrolyzed *P. sativum* (Green) and *Pisum sativum* hydrolysate (PSH; Blue)

Size exclusion chromatography (SEC) was used to highlight the different molecular weight profiles of hydrolyzed and unhydrolyzed PSH. The unhydrolyzed material (Figure 1—green chromatogram) elutes 90% of its material between 9 and 18 min whereas the hydrolyzed material (Figure 1—blue chromatogram) elutes mostly between 14 and 21 min. Based on the chromatogram, the unhydrolyzed PSH elutes faster based on the content of the higher molecular weight molecules. The unhydrolyzed PSH contains smaller molecular weight peptides and amino acids which weave through the porous beads in the column and therefore elute up to 5 min after the unhydrolyzed sample. As the only difference between the two samples was the addition (or not) of enzymes, the SEC chromatogram is indicative of successful hydrolysis whereby proteins have been digested into smaller molecular weight fragments.

### 3.2 Mass spectrometry analysis indicating the % hydrophobicity, charge, and length distribution of peptides within replicate PSH samples

**FIGURE 2:**
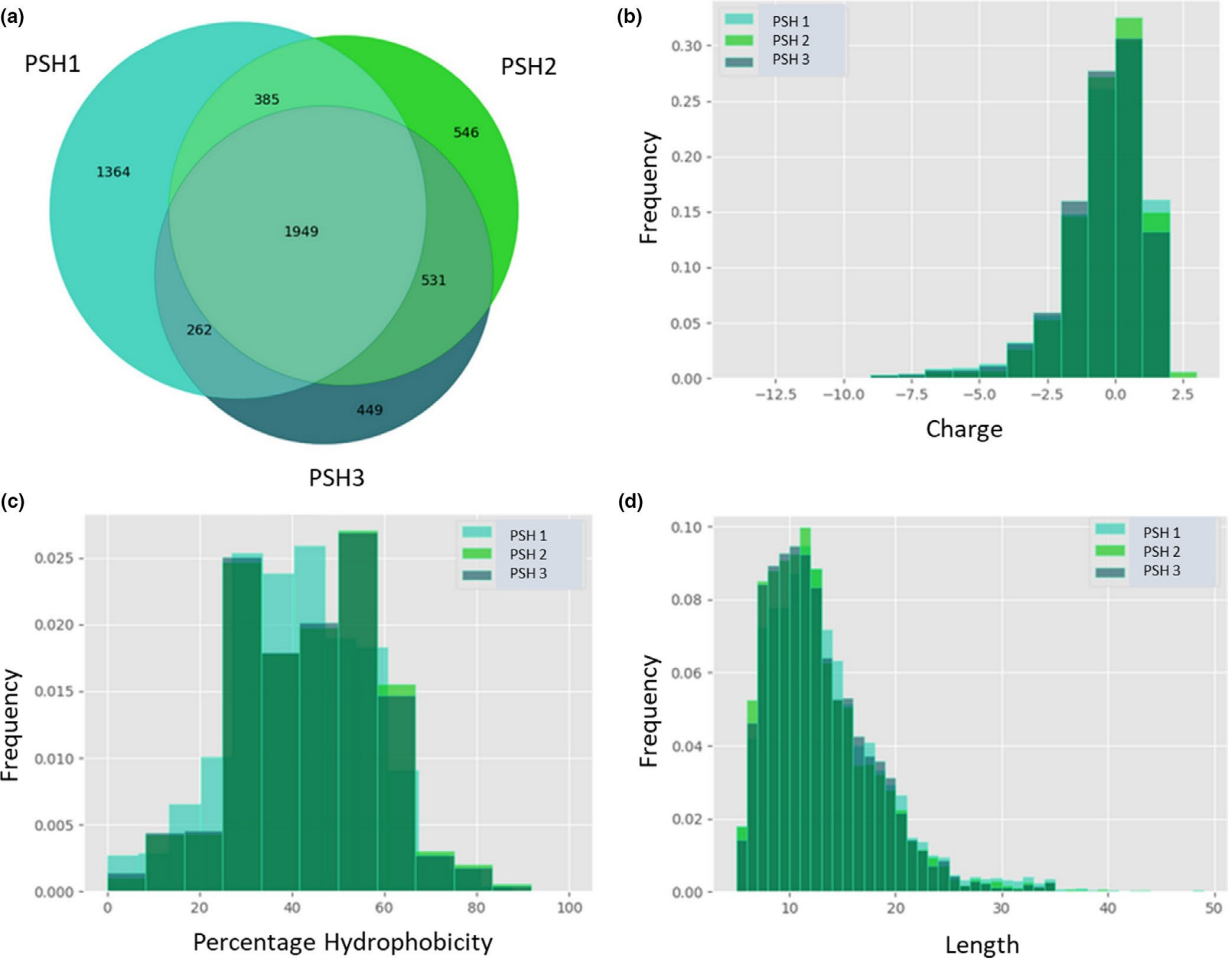
Analysis of the peptide content in *Pisum sativum* hydrolysate (PSH) 1, PSH 2, and PSH 3 hydrolyzed replicates of *P. sativum*. Histograms represent the peptides in each individual sample according to (a) a Venn diagram of the number of overlapping peptides in each replicate. In (b) Charge, (c) Percentage of Hydrophobicity and (d) Length of the three samples are shown

Mass spectrometry allowed for peptides released by the enzymatic treatment of *P. sativum* proteins to be studied. The variability in peptide content in three hydrolysate samples, PSH 1, PSH 2 and PSH 3 which were hydrolyzed on three separate days, was also determined. Studying the overlapping area of the Venn diagram in Figure 2a allowed us to investigate further only the peptides which were cleaved in a consistent manner, that is, the samples releasing identical peptides in every hydrolysis. In general, there was a good overlap between the three hydrolysates. On average, ~3,520 peptides were released posthydrolysis and ~55% (1,949 peptides) of these were common across the samples. The peptides present in the PSH samples have a net charge distribution from −2 to +2 with the majority having a −1 or neutral charge (Figure 2b). On average, the hydrophobic percentage range between 40% and 60% was where the highest frequency was observed among the three samples (Figure 2c). PSH 2 and PSH 3 were almost identical and overlapping. PSH 1 appears to have a lower hydrophobic percentage in its peptide content. Finally, the length of the peptides in the three samples, determined as molecular weight in Daltons (Da) by LC‐MS/MS, showed good consistency (Figure 2d). This indicates a robust hydrolysis procedure and good efficacy of the enzymes used, as the proteins cleaved released peptides were of very similar length, % hydrophobicity, and charge during each experimental replicate.

### 3.3 Peptides predicted to contribute to antimicrobial activity present in the overlap of the PSH samples

As each of the PSH biological triplicates was shown to have an equivalent CE concentration against *E. coli* O157:H7, we hypothesize that the activity could be due to peptides which are common among the replicates. The 1,949 overlapping peptides that are displayed in the Venn diagram in Figure 2c were listed and ranked according to four main features: net charge, length, percentage of hydrophobic residues, and their isoelectric point. The top 10 peptides that were obtained with these characteristics are shown in Table 1. These features were chosen as top classifiers for peptides which may exert antimicrobial functions based on our previous study on NuriPep 1653, which was released from the P54 protein of hydrolysis of *P. sativum* (Mohan et al., 2019). This was combined with the available information in the literature on the defining features of antimicrobial peptides important in conferring antimicrobial activity in a peptide sequence (Tossi et al., 2000).

**TABLE 1:** Top 10 peptides ranked according to charge, length, percentage of hydrophobic residues, and isoelectric point identified within the overlapping peptide regions of *Pisum sativum* hydrolysate (PSH) 1, 2, and 3 identified by MS

Peptide ID	Charge	Length	% Hydrophobicity	Isoelectric point
NuriPep 1653	4	22	50	11.26
PSPep 1	2	20	60	8.64
PSPep 2	2	20	60	8.80
PSPep 3	2	20	50	8.50
PSPep 4	2	20	50	8.50
PSPep 5	1	20	60	6.79
PSPep 6	1	19	63.16	6.79
PSPep 7	1	19	63.16	6.79
PSPep 8	1	18	44.44	8.63
PSPep 9	1	19	36.84	8.59
PSPep 10	1	18	55.56	8.59

Note

PSPep 1–10 represent the top 10 peptides identified within the overlapping section of the triplicate PSH samples which have a charge, length, percentage of hydrophobicity, and isoelectric point similar to NuriPep 1653, a previously described potent antimicrobial peptide obtained through the hydrolysis of *Pisum sativum* (Mohan et al., 2019). The longer peptides were shown to have the highest charge and hydrophobicity; however, the isoelectric point did not follow this pattern.

### 3.4 The in vitro effects of PSH against *E. coli*

The antimicrobial activity of PSH against *E. coli* was evaluated using the CE method in PBNS, thus avoiding any possible interference between ions in the buffer and the cationic peptides in the PSH. The results are shown in Table 2. All the PSH biological triplicates exhibited a CE concentration of 8 mg/ml against *E. coli*. This was considered equivalent to the minimum bactericidal concentration (MBC) usually determined as per the European Committee on Antimicrobial Susceptibility Testing (EUCAST) or the Clinical and Laboratory Standards Institute (CLSI) guidelines, as microbial growth was completely eliminated at this concentration. The unhydrolyzed material did not exhibit any bactericidal action, indicating that the whole protein material did not possess any antimicrobial activities, and these only arose after enzymatic hydrolysis, and subsequent peptide release. Leucidal liquid was active within the concentration ranges (2%–4%) previously reported by Active Micro Technologies (Active Micro Technologies, 2020).

**TABLE 2:** Complete elimination (CE) values of *Pisum sativum* hydrolysate (PSH) samples against *Escherichia coli* 0,157:H7 in vitro

Hydrolysate samples	*E. coli* O157:H7 (mg/ml)
Unhydrolyzed	>20
PSH 1	8
PSH 2	8
PSH 3	8
Leucidal liquid (control)	2%

Note

Values of PSH replicates 1, 2, and 3 required to induce bacterial clearance *via* the CE method against *E. coli* O157:H7. Unhydrolyzed *Pisum sativum* protein powder was used as a negative control.

Values shown represent the average of three independent experiments on three independent days. PSH 1–3 represent biological triplicates.

### 3.5 Antibacterial activity of PSH at inhibiting the growth of *E. coli* O157:H7 in a lettuce leaf

**FIGURE 3:**
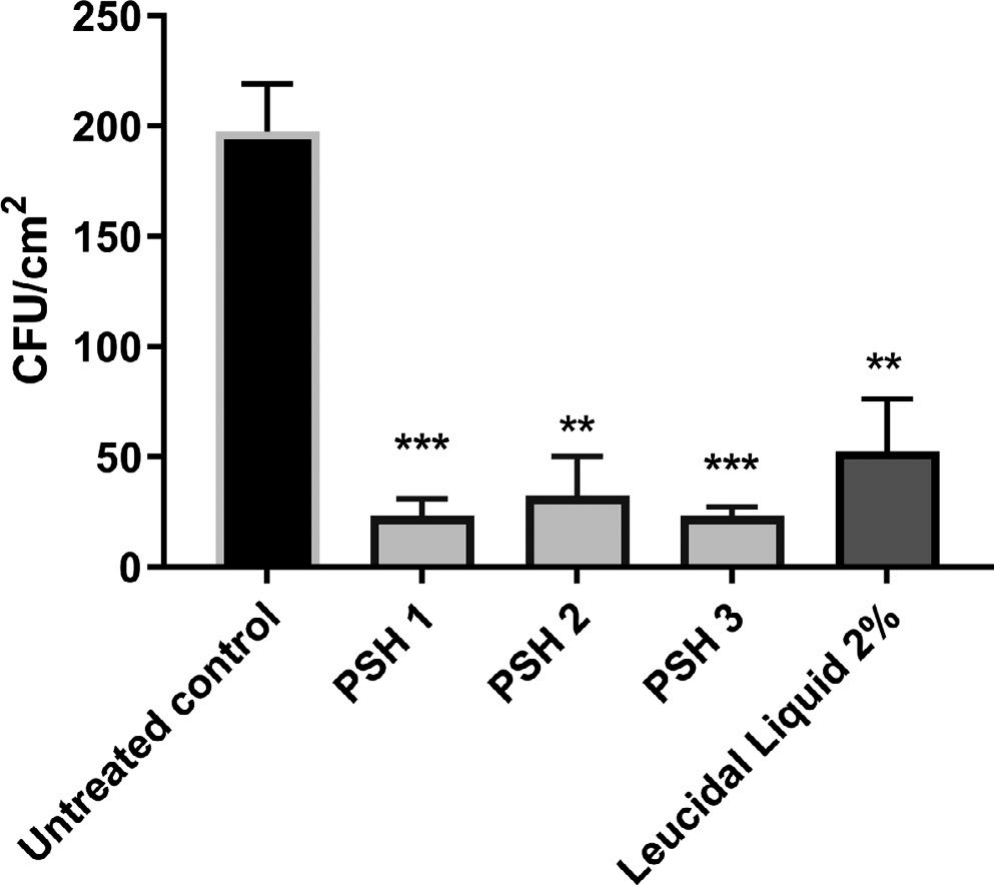
Antibacterial activity of *Pisum sativum* hydrolysate (PSH) samples at reducing populations of *Escherichia coli* O175:H7 in an infected lettuce leaf model. Reductions in the CFU/3 cm^2^ of *E. coli* recovered from the surface of an inoculated lettuce leaf were enumerated after treatment for 2 hr with PBNS (untreated control), PSH 1, PSH 2, PSH 3‐ all at 8 mg/ml and Leucidal Liquid at 2%. Bacteria were recovered on Mueller Hinton agar. Data represent the mean of three experiments performed in triplicate and is expressed as the mean ± standard deviation. Statistical analysis was performed using a one‐way ANOVA where ** *p* ≤ .01 and *** *p* ≤ .001 was considered statistically significant

The stamp on agar method was performed to evaluate surviving colonies of *E. coli* O157:H7 after treatment with PSH, Leucidal liquid, or buffer alone. As shown in Figure 3, when the three biological replicates of PSH were applied as a surface treatment spread on *E. coli* infected leaves, significant reductions (** *p* ≤ .01, *** *p* ≤ .001) in bacteria were observed after only 2 hr of treatment compared with the untreated control. In fact, the number of bacteria recovered was less than for the commercially available hydrolysate Leucidal Liquid.

### 3.6 Effects of PSH on the viability of differentiated human macrophages

**FIGURE 4:**
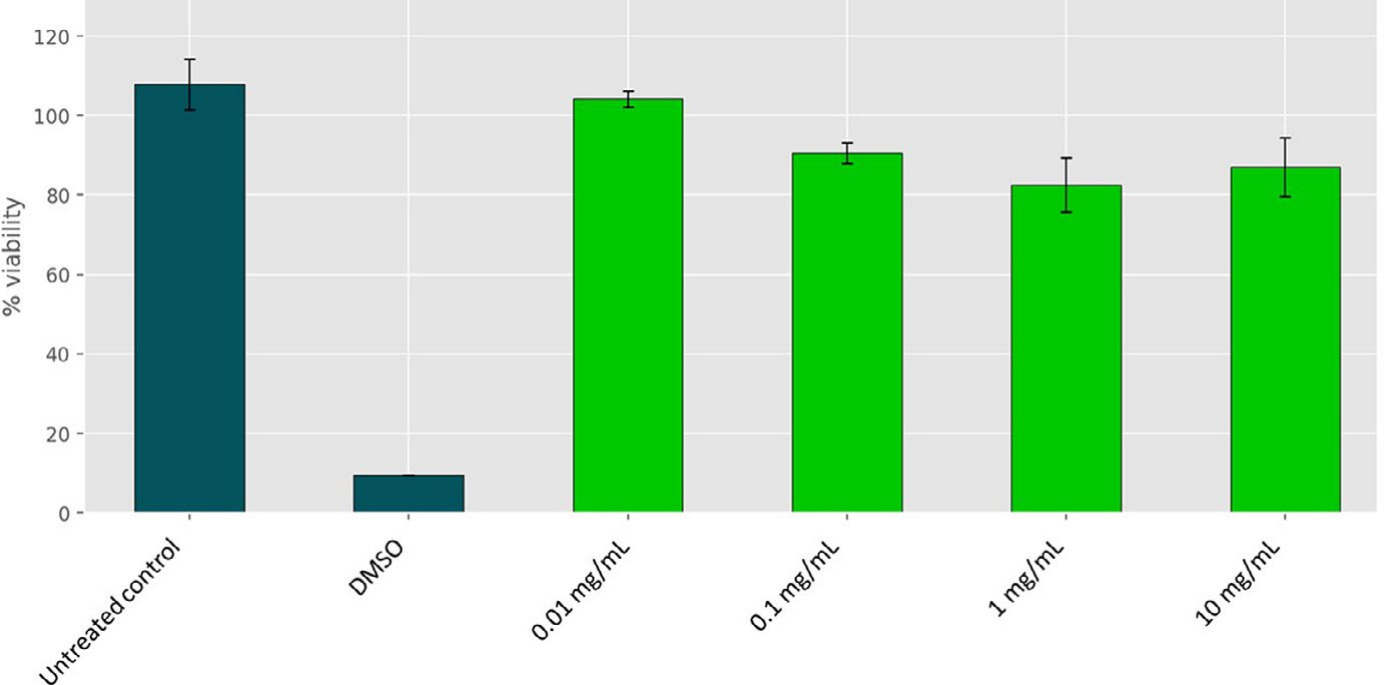
Effects of *Pisum sativum* hydrolysate (PSH) on the viability of differentiated human macrophages. Cells were differentiated with 10 mmol/L PMA for 72 hr into macrophages before treatment with the peptide for 24 hr. Cell viability was assessed by the MTT assay as per the manufacturer's guidelines. Values correspond to concentrations of PSH 1 tested at a range from 10–1,000 μg/ml. DMSO 100% and untreated cells served as controls. Data represent the mean of three experiments performed in triplicate and is expressed as the mean ± standard deviation. Statistical analysis was performed using a Student's *t* test where * *p* ≤ .05, ** *p* ≤ .01, *** *p* ≤ .001

Differentiated human macrophages were used to assess the toxicity profile of PSH and compare this to the bactericidal concentrations obtained in vitro. As shown in Figure 4, no significant adverse effects were observed on the cellular viability of the macrophages at the highest concentration of PHS 1 tested, 10 mg/ml with 85% of cells still viable. PSH 1 was chosen as a representative of all the hydrolysate samples as toxicity results observed were equivalent. DMSO performed as expected and was cytotoxic to cells. These results indicate that the concentrations of PSH required to induce bactericidal effects in vitro and to reduce the populations of *E. coli* O157:H7 on infected lettuce leaves are lower than the toxic concentration to human cells.

## 4 DISCUSSION

In this study, we present a novel bio‐preservative, PSH, produced by the enzymatic hydrolysis of *P. sativum* protein which was capable of reducing the growth of the foodborne pathogen *E. coli* O157:H7, both in vitro and on an infected lettuce leaf. Protein hydrolysis was conducted using food grade enzymes as has been commonly reported previously for plant ingredients such as wheat, rice, and pea in order to release antimicrobial peptides (Barac et al., 2012; Hou et al., 2017; Mccarthy et al., 2013). This is in contrast to the process for keratinous proteins such as those from feathers and horns which are usually hydrolyzed using acids (Hou et al., 2017; Pasupuleki & Braun, 2010). Using size exclusion high performance liquid chromatography (HPLC), we illustrated the effects of the hydrolysis procedure of *P. sativum* protein. The rapid elution of the unhydrolyzed sample in Figure 1 demonstrated avoidance with the smaller pores in the column and therefore indicated that the sample was comprised of higher molecular weight components. In contrast, the molecular weight profile of the hydrolyzed sample was shown to be made up of smaller peptides as it eluted 5 min later than the unhydrolyzed sample. Antimicrobial activity was absent in the unhydrolyzed sample, suggesting that it was the release of the peptide fragments from larger proteins which generated bioactivity and induced the bactericidal effects (Chalamaiah et al., 2018). The LC‐MS/MS investigation revealed that the length of the majority of peptides contained within the hydrolyzed PSH was between 5 and 20 amino acids. These findings allowed us to describe the peptides within PSH as fitting the length profile commonly reported for antimicrobial peptides of between 12 and 50 amino acids (Torrent et al., 2009).

Many studies describe the use of broths modeled on food products as the medium for challenge testing via the microdilution method. However, the preparation of a lettuce broth, for example, would likely release proteases which would otherwise not be released from the intact leaf (Charalampopoulos et al., 2002; Gutierrez et al., 2009; Papagianni & Papamichael, 2007). In their research, Enrique et al., 2007 and Juneja et al., 2012 describe the poor translatability of peptide antimicrobial activity from an in vitro setting into protein and lipid‐rich formulations where nonspecific binding hampers the peptide's ability to target bacterial cells. Given the difficulties associated with preserving the functionality of peptides and hydrolysates once incorporated as food ingredients, this research focused on pathogens present on the surface of food. In this way, the microdilution method was not considered to be representative of the final application as a wash or surface treatment. Instead, pieces of infected leaves were used to evaluate the potential activity of PSH as a bio‐preservative.

When applied for 2 hr at 8 mg/ml on the surface of an infected lettuce leaf, PSH was shown to significantly reduce the survival of *E. coli* O157:H7 compared with the untreated control. Furthermore, PSH outperformed the commercially available hydrolysate, Leucidal Liquid. While initially released as a cosmetic preservative, Leucidal Liquid recently gained approval for use as a food preservative (Active Micro Technologies, 2020). This is a positive indication that plant‐based protein hydrolysates are being considered as contenders to challenge chemical preservatives and are gaining momentum in the commercial market.

Rizzello et al., 2015 demonstrated the promise of a pea protein hydrolysate for the baking industry which could increase the shelf life of wheat flour by 21 days and inhibit the growth of various fungal strains. Others have described the potential preserving properties of pea hydrolysates; however, some of these studies fail to investigate the effects of their ingredients in relevant food models and instead provide details on the optimization of their hydrolysis procedures and validation of activity in in vitro systems only (Li & Aluko, 2010). Other research has focused on the use of functionalized pea protein hydrolysates as bioplastics and encapsulation vehicles for antimicrobial compounds such as nisin (Perez‐Puyana et al., 2016). However, these studies use pea hydrolysates based on their beneficial physiochemical properties such as gelling, emulsifying, and foaming activities rather than exploring any inherent bioactive properties. The contribution of this study is therefore not only to describe the production of a novel bio‐preservative using enzymatic hydrolysis, but to validate its activity in an in vitro and food model setting and highlight some of the features of the peptides which are likely responsible for the antimicrobial effects.

Protein hydrolysates contain networks of peptides which often function synergistically to exert a wide range of bioactivities (Yoo et al., 2014). Given the well‐described, membrane active, mode of action of cationic antimicrobial peptides, it is likely that some of the peptides within the PSH exert their activities through electrostatic attraction, irreversible permeabilization, and lysis (Yeaman & Yount, 2003). Using knowledge developed in a previous study on identifying peptide characteristics and motifs associated with conferring antimicrobial activity, the MS output, posthydrolysis, was screened for peptides which were likely to contribute to the antimicrobial activity of the PSH (Mohan et al., 2019). While no peptides were identified bearing as high a charge (+4) or isoelectric point (11.26) as our previously reported *P. sativum* peptide, NuriPep 1653, cationic peptides of similar length and percentage of hydrophobic residues of 40%–50% were identified as shown and ranked in Table 1. Our findings are in line with those previously reported whereby the percentage of hydrophobic residues in antimicrobial peptides is commonly around 50% (Huang et al., 2010). We hypothesize that these peptides may contribute to the functionality of the PSH and could be investigated for their individual antimicrobial activities and potential use in higher value industries such as pharmaceuticals where profit margins are greater and can justify the high peptide production costs. An important limitation identified during this study was the necessary use of SPE, posthydrolysis, to remove salts and subsequently preserve bioactivity. The disadvantages of the SPE step were twofold. Firstly, the final yield of PSH was low, which limited the sample size of lettuce available for testing. Considering this, our sample size was modeled on the size of lettuce pieces observed in bagged iceberg lettuce as opposed to whole leaves. Secondly, while SPE is commonly reported in research as a step in hydrolysate preparation from various sources, it is undesirable during commercial production as it significantly adds to cost (Hansen et al., 2020). Membrane filtration and ultrafiltration are used regularly in the industry as cost‐effective, scaled‐up methods to desalt protein hydrolysates (Karimi et al., 2020). While this research provides a proof‐of‐concept, alternative salt extraction methods would need to be considered if PSH were to be investigated in a larger food model study or brought forward for commercial application to compete with very cheap chemicals like chlorine.

One of the constraints of using hydrolysates in food products is the possibility of changes in the appearance and color of the products. Some studies have described methods to decolorize and reduce haze in hydrolysates such as by adding charcoal powder (Hou et al., 2017). This may not be necessary for PSH since the visual appearance and odor of the treated lettuce did not change; however, a longer exposure time with the PSH would be necessary to confirm this. Bitterness can be encountered with protein hydrolysates, in particular those with low molecular weight peptides, bulky hydrophobic amino acids at the C terminus, and basic amino acids at the N terminus. These have been implicated in the generation of adverse flavors (Hou et al., 2017; Lafarga & Hayes, 2016; Maehashi & Huang, 2009). Changes in the organoleptic profile of the lettuce after the addition of PSH were not assessed in the present study; however, protein hydrolysates are likely to be better tolerated and present less adverse impacts on taste when applied to food in comparison to other natural preservatives, such as essential oils (Sultanbawa, 2011). Natural oils have also been reported as potentially being able to alter the gut microbiome (Dorman & Deans, 2000). This is not a concern with peptides and protein hydrolysates as they are subjected to natural digestion and absorption in the GI tract, limiting antimicrobial activity to the actions on food prior to consumption (Korhonen, 2009).

The safety of various artificial preservatives and washing treatments has come into question in recent years. PSH did not cause any cytotoxic effects at concentrations required to kill bacteria in vitro or on the lettuce. In general, protein hydrolysates have been reported as well tolerated and nontoxic, similar to the intake of intact proteins which have a history of safe consumption over hundreds of years and are subject to natural breakdown in the body, as described above (Schaafsma, 2009).

While limited toxicity is an advantage of protein hydrolysates, variability can sometimes occur based on seasonal, biological, and chemical changes in the natural source material, which can limit their use. Despite this, little batch to batch variation was observed as all three independent PSH samples 1, 2, and 3 gave equivalent CE concentrations, performed well in the infected lettuce leaf model, and showed the same cytotoxicity profile in human cells. MS analysis of the peptides in each sample showed more than a 50% overlap in constitutively released peptides after hydrolysis. Based on these findings, we hypothesize that these commonly occurring peptides are the ones responsible for activity.

Most of the currently employed decontamination methods for fresh produce are not favorable for a number of reasons, but most importantly their safety to humans and the environment. Protein hydrolysates, such as PSH, represent an environmentally friendly, plant‐based means to preserve food using the naturally occurring bioactive components found within food proteins. While this study focuses on reducing *E. coli* O157:H7 on a lettuce leaf model, future work could explore how this novel, natural bio‐preservative may be applied to protect against other food pathogens and expand its potential to the preservation of meats, seafood, and dairy products. Therefore, this work opens the door to a “from food—for food” preservation approach for food products which will hopefully lead to the discovery of many more bioactive hydrolysates with applications in the food industry and beyond.

## CONFLICT OF INTERESTS

Zorgani A, Earley L, Chauhan S, Trajkovic S, Savage J, Adelfio A, and Khaldi N were employed by the company Nuritas Limited. The remaining authors declare that the research was conducted in the absence of any commercial or financial relationships that could be construed as a potential conflict of interest.

## AUTHOR CONTRIBUTIONS

**Niamh Maire Mohan:** Formal analysis (lead); Investigation (lead); Methodology (lead); Validation (lead); Visualization (lead); Writing‐original draft (lead); Writing‐review & editing (lead). **Amine Zorgani:** Formal analysis (supporting); Supervision (supporting); Writing‐review & editing (supporting). **Leah Earley:** Formal analysis (equal); Methodology (equal); Writing‐review & editing (equal). **Sweeny Chauhan:** Methodology (equal); Writing‐review & editing (supporting). **Sanja Trajkovic:** Methodology (equal); Writing‐review & editing (supporting). **John Savage:** Formal analysis (equal); Methodology (equal); Writing‐review & editing (supporting). **Alessandro Adelfio:** Formal analysis (equal); Methodology (equal); Writing‐review & editing (supporting). **Nora Khaldi:** Conceptualization (equal); Funding acquisition (equal); Investigation (equal); Methodology (equal); Resources (equal); Supervision (equal); Writing‐review & editing (supporting). **Marta Martins:** Conceptualization (equal); Formal analysis (equal); Funding acquisition (equal); Investigation (equal); Methodology (equal); Project administration (equal); Resources (equal); Supervision (equal); Visualization (equal); Writing‐review & editing (equal).

## Supporting information

Table S1
