# Supplementary material for: Preservatives from food—For food: Pea protein hydrolysate as a novel bio‐preservative against Escherichia coli O157:H7 on a lettuce leaf
Source: Food Sci Nutr. 2021 Sep 8;9(11):5946–58. doi: 10.1002/fsn3.2489 (PMC8565202; doi:10.1002/fsn3.2489)
Supplement: Supplementary file 1 — Table S1 [file FSN3-9-5946-s001.docx]

**Supplementary Table 1: Advantages and disadvantages of currently used washing treatments in food preservation.**

| **Washing treatment** | **Advantages** | | **Disadvantages** | **References** |
| --- | --- | --- | --- | --- |
| Chlorine | - Highly efficient at low concentrations - Cost effective - Easy to use - Short contact time | - It can release toxic gas and corrode equipment below pH 6 - Initial organic load can reduce efficacy - If bound to organic material it can become highly toxic to humans and the environment | | (Petri *et al*., 2015)  (Jongen, 2005)  (Beuchat & Ryu, 1997)  (Siroli, 2014)  (Gil *et al*., 2009) |
| Acid treatment | - Superior activity to chlorine - No harmful side effects - Environmentally safe - GRAS status | - Potential sensory changes - Long contact times necessary | | (Rico *et al.*, 2007)  (Siroli, 2014)  (Tirpanalan *et al*., 2011) |
| Hydrogen peroxide | - Bactericidal and sporicidal - Environmentally friendly - GRAS status as surface decontaminant | - Not permitted as washing additive - Major sensory changes in certain products | | (Lee *et al*., 2014)  (Siroli, 2014)  (Beuchat & Ryu, 1997) |
| Ozone | - Bactericidal and sporicidal - Environmentally friendly - Can degrade certain pesticides - GRAS status - Works over a broad pH range on various food types | - Potential health risk of those in processing facilities - Potential sensory changes - High capital investment - Accelerated erosion of equipment | | (Jongen, 2005)  (Guzel- Seydim *et al*., 2004)  (Brodowska *et al.*, 2018) |
| Calcium wash | - Can improve texture - Can increase calcium content of final product | - Can have a bitter taste - Minimal studies conducted on antimicrobial ability | | (Martin-Diana *et al*., 2007) |
| Electrolysed water | - More effective against pathogens than chlorine and ozone - Can neutralise toxins - Highly stable | - Can cause discolouration - Requires a constant supply of hydrogen chloride and ions - Accelerated corrosion of equipment | | (Hsu, 2003)  (Petri *et al*., 2015) |

**Legend.** GRAS – Generally Regarded As Safe.
